# Global database of FRP-to-masonry bond strength tests

**DOI:** 10.1016/j.dib.2018.08.111

**Published:** 2018-08-30

**Authors:** J. Vaculik, P. Visintin, N.G. Burton

**Affiliations:** The University of Adelaide, Australia

## Abstract

Quantifying the bond strength between fibre-reinforced-polymer (FRP) composites and substrates is essential to the design of FRP retrofit systems. This paper collates a database of 1583 individual tests across 56 published experimental campaigns investigating the FRP-to-masonry bond strength through shear pull-tests. Included in the database is all available information characterizing the test arrangement, geometric and mechanical properties of the constituents, as well as the failure load and failure mode.

**Specifications table**

**Table:** 

Subject area	*Engineering*
More specific subject area	*Strengthening structures, retrofitting*
Type of data	*Tables*
How data was acquired	*Literature review*
Data format	*Analysed*
Experimental factors	*N/A*
Experimental features	*N/A*
Data source location	*Italy (64%), Portugal (7%), Germany (6%), Poland (5%), Australia (5%); remaining 12% from Canada, New Zealand, Turkey, Greece, Spain, Iran, US, France*
Data accessibility	*With this article*
Related research article	Vaculik, J., Visintin, P., Burton, N.G., Griffith, M.C., Seracino, R. (2018) State-of-the-art review and future research directions for FRP-to-masonry bond research: Test methods and techniques for extraction of bond-slip behaviour. *Construction and Building Materials, In Press*.

**Value of the data**

• Quantification of bond strength is essential to the design of FRP retrofit systems for strengthening structures.
• The database presented collates the results of 1583 bond strength tests from 56 published studies.
• The data can be used as the basis for the calibration of new bond strength design rules.
• The data can be used as a benchmarking dataset for comparing new and existing design rules.

## 1 Data

Through an extensive literature review, an experimental database of 1583 individual shear pull-tests on masonry specimens was compiled from across 56 published studies [1], [2], [3], [4], [5], [6], [7], [8], [9], [10], [11], [12], [13], [14], [15], [16], [17], [18], [19], [20], [21], [22], [23], [24], [25], [26], [27], [28], [29], [30], [31], [32], [33], [34], [35], [36], [37], [38], [39], [40], [41], [42], [43], [44], [45], [46], [47], [48], [49], [50], [51], [52], [53], [54], [55], [56].

### 1.1 Description of a pull-test

**Fig. 1:**
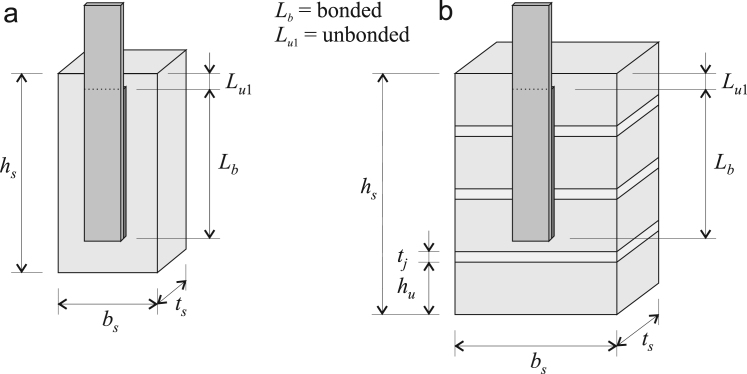
Generic pull-test specimen and associated geometric definitions for (a) unit prism, and (b) masonry prism.

A generic pull-test specimen consists of a fibre-reinforced-polymer (FRP) composite plate adhesively bonded to a substrate prism over a particular lap length. The substrate prism can be either a *unit prism* consisting of a single brick or block, or a *masonry prism* consisting of individual units bonded together using mortar joints. Both of these are shown in Fig. 1.

A test is performed by applying an increasing tensile force to the FRP plate until the plate eventually debonds from the prism. Typical debonding failure involves the detachment of the plate along with a layer of the substrate material. Alternate failure modes (other than substrate debonding can include FRP rupture, failure at the adhesive, prism material failure (compression, tension or shear), or a combination of these.

The test results summarised in the compiled database include the maximum load and a description of the observed failure mode. Note that the various possible forms of instrumentation can include the measurement of strain along the plate using strain gauges, slip (displacement) between the plate and the prism, or full-field deformation by techniques such as digital image correlation. However, as the focus of the database is the bond strength, these are beyond the scope of the data.

### 1.2 Scope of tests in the database

#### 1.2.1 Substrate materials

Tested substrate materials include clay brick, limestone, tuff, concrete block, calcium silicate brick, sandstone, and mortar specimens. Data for each test includes the mechanical properties of the substrate material including its compressive and tensile strength. The tensile strength is further subcategorised in terms of the type of test performed as either direct, flexural, splitting, or unspecified.

#### 1.2.2 FRP materials

**Fig. 2:**
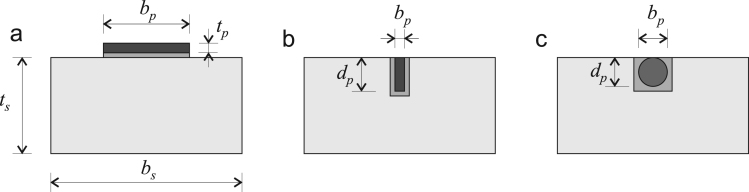
Cross section and geometric definitions in (a) an externally-bonded retrofit, (b) NSM retrofit using a rectangular strip, and (c) NSM retrofit using a circular rod.

The database covers both externally-bonded (EB) and near-surface-mounted (NSM) retrofits, as shown in Fig. 2. The different possible reinforcement shapes include EB sheets (a.k.a. fabrics) installed by wet lay-up (see Fig. 2a), and pre-formed rectangular strips and round bars used in NSM applications (Fig. 2b and c).

Composite materials include carbon FRP (CFRP), glass FRP (GFRP), basalt FRP (BFRP), steel-reinforced polymers (SRP), aramid FRP (AFRP), and natural flax FRP. The majority of tests use epoxy adhesive but some also use polyurethane or cementitious adhesive.

#### 1.2.3 Test arrangements

**Fig. 3:**
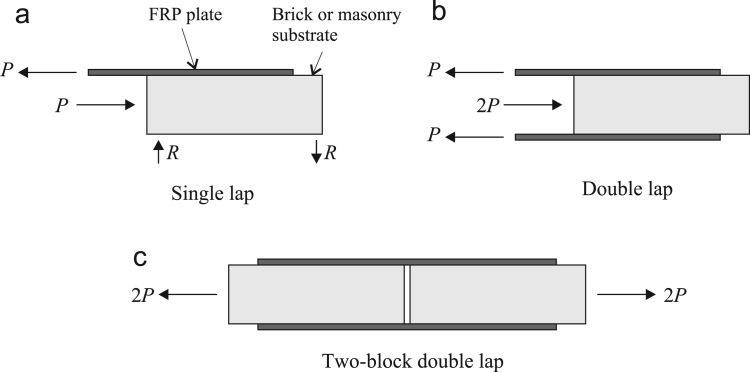
Alternate test arrangements including: (a) single lap, (b) double lap, and (c) two-block double lap.

Tests within the database are split approximately evenly between single-lap and double-lap arrangements, with the latter being further subcategorised into a single block and a double block variant. Each of these are shown in Fig. 3. Note that for double-lap arrangement the reported ultimate load corresponds to the load in a single lap, *P* as shown in Fig. 3.

The majority of tests in the database use monotonic loading. However, a small number of tests used cyclic loading comprising separate loading, unloading and reloading phases.

### 1.3 Data inclusion/exclusion criteria

The database includes only tests conducted under standard conditions, that is, where the plate is bonded directly to the masonry substrate and subjected to quasi-static loading. The data therefore excludes the following:

• Specimens subjected to effects such as temperature and moisture [5], [25], [29], [33], [41], [46], [51], [52];
• Tests in which additional anchorage between the FRP and substrate was provided. For example those with nails, fans or cogs [9], [18], [24], [30], [32], [55];
• Tests in which the FRP was bonded to plaster instead of directly to the masonry substrate [9], [39], [42], [55], [56];
• Non-quasi-static loading conditions such as impulse loads [54]; and
• Plates manufactured from textile-reinforced mortars or fibre-reinforced cementitious mortars.

These exclusion criteria were applied to individual tests, and as such, control specimens found in the aforementioned studies are still included. Note that some studies have intentionally opted not to report the failure load for tests where the mode of failure was not by interfacial debonding—such tests are still included as long as the mode of failure was reported. Additionally, where the same tests were identified to have been reported in multiple sources, they are only included once.

The database also includes tests with the following special conditions, which are specifically identified in the database:

• Tests with confining pre-compression (30 tests) [13], [19];
• Curved specimens (10 tests) [18]; and
• Specimens that were repaired after an initial test and re-tested (98 tests) [34], [40], [50].

### 1.4 Description of information provided in the database

The compiled database is provided as a CSV (comma separated variable) spreadsheet in Supplementary Material accompanying this article. Table 1 summarises the data provided for each individual pull-test. Note that fields entered as ‘-‘ mean that the data is either not relevant to the particular test or that it was not specified in the original paper.

**Table 1:** Definition of columns within the database.

Col.	Header	Description
1	Reference	Original reference author and year
2	Test ID	Test identifier
3	Substrate Material (Family)	Substrate material family: ׳Clay Brick׳, ׳Limestone׳, ׳Tuff׳, ׳Sandstone׳, ׳Concrete Block׳, ׳Calcium Silicate Brick׳, or ׳Mortar׳
4	Substrate Material (Descriptive)	More detailed description of the substrate material. Used also to distinguish between unique substrates reported in the original reference.
5	fuc (MPa)	Compressive strength of substrate unit
6	fut,dir (MPa)	Tensile strength of substrate unit from direct tension test
7	fut,flex (MPa)	Tensile strength of substrate unit from flexural test
8	fut,split (MPa)	Tensile strength of substrate unit from splitting test
9	fut,unspec (MPa)	Tensile strength of substrate unit from unspecified type of test
10	Prism	Prism type as either ׳Unit׳ (individual unit only) or ׳Masonry׳ (units + mortar)
11	bs (mm)	Width of prism
12	ts (mm)	Depth of prism (perpendicular to bonded face)
13	hs (mm)	Height of prism (parallel to FRP axis)
14	hu (mm)	Height of individual unit measured parallel to FRP axis (applies to masonry prisms only)
15	tj (mm)	Thickness of mortar joints measured parallel to FRP axis (applies to masonry prisms only)
16	Cored Units	Denotes whether units are cored
17	Through Perpends	Denotes whether FRP passes through perpend joints
18	Has Precomp	Denotes whether specimen has precompression
19	Is Repaired	Denotes whether specimen is a previously tested and repaired specimen
20	Other Special Features	Any special features other than those listed in the previous several columns
21	Laps	Number of tested laps. Can be ׳Single׳, ׳Double׳ or ׳2-Block Double׳ (i.e. 4 laps)
22	FRP Material	FRP material: CFRP, GFRP, BFRP, SRP, AFRP or Flax
23	FRP Shape	Shape of FRP material: ׳Sheet׳ (wet lay-up fabric), ׳Strip׳ (rectangular) or ׳Rod׳ (round)
24	FRP Configuration	Type of retrofit: ׳EB׳ (externally-bonded) or ׳NSM׳ (near-surface-mounted)
25	fpu (MPa)	Ultimate tensile stress of FRP
26	Ep (MPa)	Modulus of elasticity of FRP
27	bp (mm)	Width of FRP plate (in both EB and NSM)
28	tp (EB) (mm)	Thickness of FRP plate (applies to EB only)
29	dp (NSM) (mm)	Depth of the FRP plate (applies to NSM only)
30	Lb (mm)	Bonded length of plate (along FRP axis)
31	Lu1 (mm)	Unbonded length provided at the loaded end of specimen
32	Adhesive type	"Epoxy" or "Cementitious"
33	Loading	"Monotonic" or "Cyclic"
34	Pmax (kN)	Failure load
35	No. Reps	Number of repetitions (denotes whether given Pmax is given as average of multiple)
36	cov(Pmax)	Coefficient of variation in Pmax (applies only to cases where Pmax is given as average of multiple)
37	Failure Mode	Reported failure mode(s): "Substrate Debonding", "FRP rupture", "Adhesive failure", "Prism failure", or a combination.
38	Comment	Provides comment regarding any provided information that differs from the original paper
